# First-in-Class Phosphorus Dendritic Framework, a Wide Surface Functional Group Palette Bringing Noteworthy Anti-Cancer and Anti-Tuberculosis Activities: What Lessons to Learn?

**DOI:** 10.3390/molecules26123708

**Published:** 2021-06-17

**Authors:** Serge Mignani, Jérôme Bignon, Xiangyang Shi, Jean-Pierre Majoral

**Affiliations:** 1 Laboratoire de Chimie et de Biochimie Pharmacologiques et Toxicologique, PRES Sorbonne Paris Cité, CNRS UMR 860, Université Paris Descartes, 45, Rue des Saints Peres, 75006 Paris, France; 2 CQM-Centro de Química da Madeira, MMRG, Campus da Penteada, Universidade da Madeira, 9020-105 Funchal, Portugal; 3 Institut de Chimie des Substances Naturelles du CNRS, 91198 Avenue de la Terrasse, CEDEX, Gif-sur-Yvette, 91190 Paris, France; Jerome.BIGNON@cnrs.fr; 4 College of Chemistry, Chemical Engineering and Biotechnology, Donghua University, Shanghai 201620, China; 5 Laboratoire de Chimie de Coordination du CNRS, 205 Route de Narbonne, BP 44099, CEDEX 4, 31077 Toulouse, France; 6 LCC-CNRS, Université de Toulouse, CNRS, 31077 Toulouse, France

**Keywords:** nanomedicine, phosphorus dendrimers, phosphorus dendrons, anti-cancer agents, anti-tubercular agents

## Abstract

Based on phenotypic screening, the major advantages of phosphorus dendrimers and dendrons as drugs allowed the discovery of new therapeutic applications, for instance, as anti-cancer and anti-tuberculosis agents. These biological activities depend on the nature of the chemical groups (neutral or cationic) on their surface as well as their generation. As lessons to learn, in the oncology domain, the increase in the generation of metallo-dendrimers is in the same direction as the anti-proliferative activities, in contrast to the development of polycationic dendrimers, where the most potent anti-tuberculosis phosphorus dendrimer was observed to have the lowest generation (G0). The examples presented in this original analysis of phosphorus dendrimers and dendrons provide support for the lessons learned and for the development of new nanoparticles in nanomedicine.

## 1. Introduction

**Figure 1:**
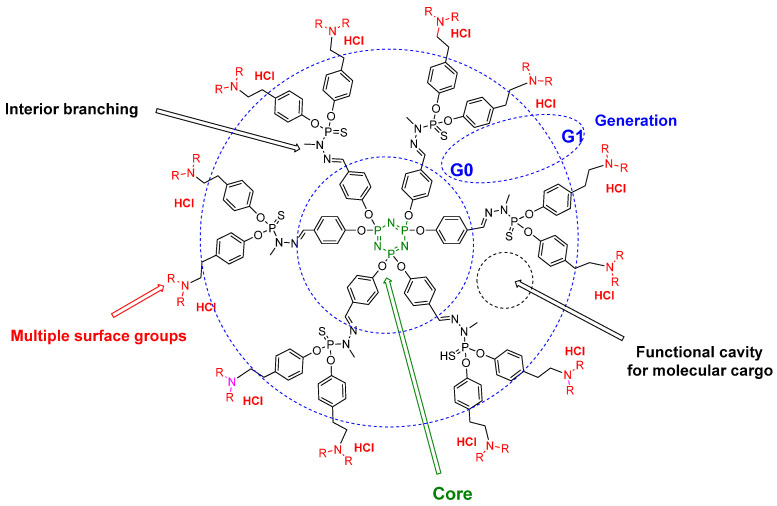
Two-dimensional chemical structures of phosphorus dendrimers: generations 0 and 1.

Biocompatible dendrimers incorporating phosphorus account for over 100 families of dendrimers (globular tree-like structures) [1], and within the dendrimer space concept, as a continuum chemical space defined by us in 2013 [2]. The introduction of phosphorus allows the versatile construction of a large variety of useful components of the dendrimers, including the core, branching units, internal branches, backbones and surface moieties [3]. Synthesis of highly branched phosphorus dendrimers with well-defined size, shape, molecular weight and monodispersity versus the non-globular structure of linear polymers of the same molecular weight can be easily performed under mild reaction conditions, releasing by-products such as water and sodium salts that can be removed without the use of any sophisticated techniques. A stepwise repetitive reaction sequence is used, and generally the final phosphorus dendrimers are obtained in good overall yields, whatever their generation (termed Gn). As dendrimers have a symmetric repetition of branches (Gn) starting from the core, and exhibit three-dimensional morphology of nanometer-scale size, they can be classified based on their generation number (G0–G4, etc.). Notably, the salient advantageous features of dendritic polymers, which include dendrimers and linear polymers, are as follows: highly compact/globular structure allowing precise control of molecular weight and monodispersity, along with reproducible pharmacokinetic (PK)/pharmacodynamic (PD) behaviors; and high structural control of branches (topology); and versatility in design and modification of terminal end groups. One useful method for the synthesis of phosphorus dendrimers starting from commercially available hexachlorocyclotriphosphazene is based on the repetition of two successive quantitative reactions, allowing phosphorhydrazone linkages (branches) and involving firstly the condensation at low temperature of aldehyde moieties bearing the branches of dendrimers by (1-methylhydrazine)phosphorothioic dichloride, followed by condensation of the two resulting chlorine atoms by large functionalized phenols, including fluorophoric molecules for in cellulo and in vivo purposes, as well as other functional groups. The incorporation of a large number of types of functional groups (e.g., amines, hydroxyl groups, carboxylic groups, phosphonates, phosphates, or complexed metals) symetrically or non-symetrically distributed on the surface of phosphorus dendrimers provides a wide palette of biological applications, including imaging applications, based on precise control of the dendritic framework enabling the final desired features to be obtained [3]. Figure 1 depicts the two-dimensional chemical structures of an example of G0 and G1 phosphorus dendrimers.

Remarkably, this synthetic method allowed us to prepare phosphorus dendrimers up to G12 [4]. The advantage of developing phosphorus dendrimers is the ability to rapidly increase the number of generations with the minimum of reaction steps, thus lessening the probability of introducing structural defects into the dendritic framework [3]. Nevertheless, several strategies have been implemented to solve this issue in order to prepare high-generation dendrimers. For instance, click chemistry has been used to prepare sophisticated dendrimers [5]; accelerated synthesis of phosphorus-containing dendrimers from orthogonal systems has been developed using two-branched AB_5_ and CD_5_ monomers for condensation and Staudinger reactions [6]; broadening of the phosphorus dendrimer design can be highlighted by the preparation of hybrid phosphorus–viologen dendrimers, as well as macromolecular asterisks with several types of cores, including 1,6-diphosphine [7], phosphonate-phosphine [8] and cage diphosphine [9]; and onion peel dendritic structures [10] and Janus dendrimers [11] have also been developed.

For drug delivery (majority of studies), drug products (e.g., small molecules, large molecules) can be either encapsulated inside the free space within the dendritic architecture of dendrimers with hydrophilic and/or hydrophobic interactions, or chemically attached or physically adsorbed onto the dendrimer surface (e.g., siRNA) [12,13]. An alternative strategy is that dendrimers in general and phosphorus dendrimers in particular can be developed as drugs themselves [14]. To date, several examples have been described: PAMAM dendrimers [15], carbosilane dendrimers [16,17] and phosphorus dendrimers. As advocated by Frechet and Szoka et al. [18] and Khandare and Haag et al. [19], the success of dendrimers in nanomedicine, both as nanocarriers and as drugs, is strongly related to their in vivo biocompatibility. The challenges and limitations during active research development, such as clinical translation factors and the challenge of current dogmas of carefully assembled dendrimers as first-in-class and best-in-class, were recently emphasized [20,21,22,23]. Nanoparticles in general, and dendrimers in particular, that encapsulate or conjugate biologically active molecules or drugs can be considered best-in-class when used clinically. This strategy is based on the host–guest properties of dendrimers [24]. Indeed, this nano formulation improved the physicochemical (pharmacokinetic/pharmacodynamic) properties as well as the biological activity of the considered drug, with a new nanoparticle type (e.g., phosphorus dendrimers) and consequently a new science (e.g., molecular mechanism of action) that in itself can be considered to be first-in-class (vide infra). The objectives and strengths of this less-trodden drug development path are the treatment of unprecedented patient outcomes in ways that are superior to existing treatments.

Importantly, unique success in the translation process from bench to clinic and then to market with first-in-class L-lysine dendrimers was achieved by Strapharma (Australia) with the development of VivaGel^®^ (SPL7013) [25], an antiviral/antibacterial drug used for the prevention of bacterial vaginosis [26] and also recently against COVID-19 infection [25]. These noteworthy examples of case studies encourage everybody to enter this therapeutic realm for the development of new biocompatible dendrimers as drugs to tackle difficult diseases. Today, we are convinced that only part of the iceberg of tailored phosphorus dendrimer design as drugs has emerged, and that many fascinating therapeutic perspectives and new opportunities have yet to be uncovered.

The main therapeutic domains developed by J.-P. Majoral and A.-M. Caminade are as follows: anti-inflammatory [27,28], anti-tuberculosis [29], against Alzheimer’s disease [30], antiprion [31] and anti-cancer [32,33,34,35]. The theragnostic realm in oncology [36] has also been developed using phosphorus dendrimers and dendrons [37,38,39].

It is generally thought that the nature of the surface chemical units (e.g., polycationic, polyanionic and neutral moieties) of dendrimers plays a crucial role in both the in vitro and in vivo therapeutic applications of these nanoparticles. Phosphorus dendrimers are no exception to this rule. Modification of the nature of the groups on the surface of phosphorus dendrimers crucially influences the in vitro and in vivo biological activities and delineates new ‘druggable’ clusters within the chemical space.

The purpose of this original manuscript is to review and analyze our recent first-in-class development of neutral and cationic phosphorus dendrimers and dendrons, active per se, in the oncology and anti-tuberculosis domains. Importantly, the nature of the surface moieties (cationic and neutral) and the generation of dendrimers and dendrons play a crucial role in the in vitro and in vivo biological activities. We will clearly show that the increase of generation in Cu(II) and Au(III) moieties of metallodendrimers increases the biological activities as anti-cancer agents, whereas the decrease of generation in polycationic dendrimers increases the anti-tuberculosis activity in vitro and in vivo. Additionally, we will analyze in more detail the respective molecular mechanisms of action related to the generations of dendrimers and dendrons. Importantly, all these biological phosphorus dendrimers and dendrons were found using phenotypic screening. These observations have never been published before, and therefore constitute a lesson learned.

## 2. Neutral Phosphorus Dendrimers as Anti-Cancer Agents

**Figure 2:**
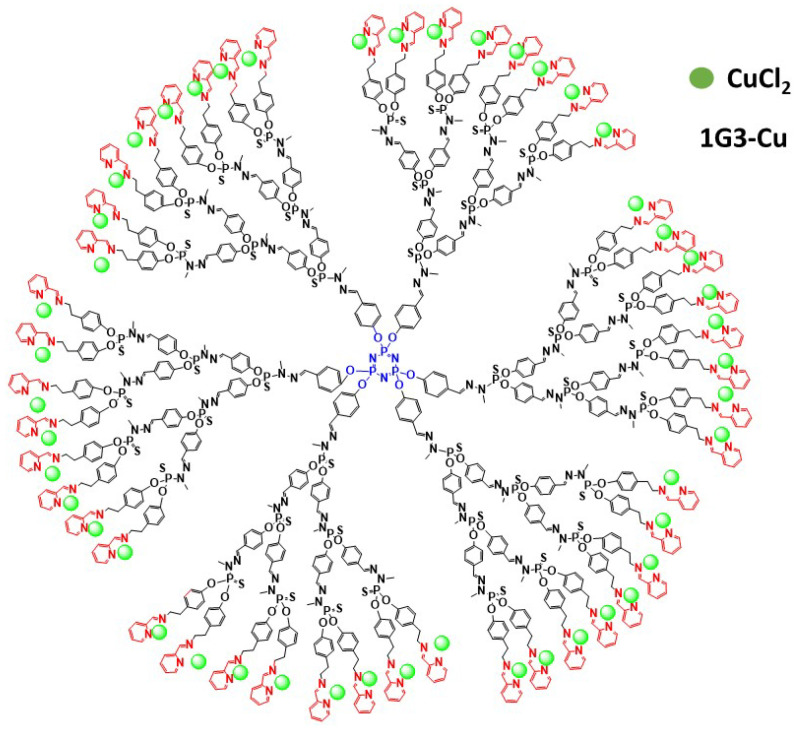
Schematic depiction of 2D chemical structure of **1G3-Cu**.

Oncology is the main therapeutic realm in which nanoparticles, including dendrimers, are used. We started our investigations to find and develop new phosphorus dendrimers as anti-cancer agents by introducing multivalent chemical groups including Cu(II), Au(III) and Cu(II)–Au(III) on the surface of phosphorus dendrimers. The selected strategy was based on phenotypic screening against two different tumor cells, including solid tumor, epidermoid carcinoma KB and leukemia promyelocytic HL60 tumor cell lines. The ligand types were developed, but only the *N*-(4yridine-2-ylmethylene)ethanamine series showed potent anti-proliferative activities against KB and HL60 tumor cell lines. The most potent Cu(II)-containing phosphorus dendrimers (G3: named **1G3-Cu**, with 48 terminal groups; Figure 2) were tested against a panel of tumors, including human glioblastoma astrocytoma epithelial-like U87-MG, ovarian carcinoma OVCAR8, hormone-responsive breast cancer MCF7 and human colon cancer HCT116. The half-maximal inhibitory concentration (IC_50_) ranges between ~0.5 and 1 µM. Importantly, a good safety ratio (~4–10-fold versus tumor cell lines) was observed against non-cancer cell lines, which include MCR5 (proliferative human lung fibroblast) and quiescent endothelial progenitor cells (EPC). In addition, each terminal group on the surface of phosphorus dendrimers contributes to the global anti-cancer activity. Interestingly, as a new molecular mechanism of action to fight cancers, we showed that **1G3-Cu** strongly translocates the pro-apoptotic Bax protein, promoting the release of apoptosis-inducing factor and, finally, activates the caspase-independent apoptotic pathway. **1G3-Cu** exposure also resulted in a decrease of intracellular ATP concentration, thus promoting cell apoptosis.

To overcome cross-therapy drug resistance in cancers, the strategy of a combination of drugs using different modes of action was used [40]. The anti-proliferative activity of **1G3-Cu** in a cocktail of several canonical cytotoxic drugs used in chemotherapy, such as camptothecin (topoisomerase I inhibitor), cisplatin (DNA cross-linker/intercalator), taxol (microtubule stabilizer), MG-132 (proteasome inhibitor) and doxorubicin (DNA intercalator/topoisomerase II inhibitor), is an example: no additive outcome with camptothecin and cisplatin was observed, but there was an additive response with paclitaxel and MG-132 and also a very interesting synergy with doxorubicin [32].

**Figure 3:**
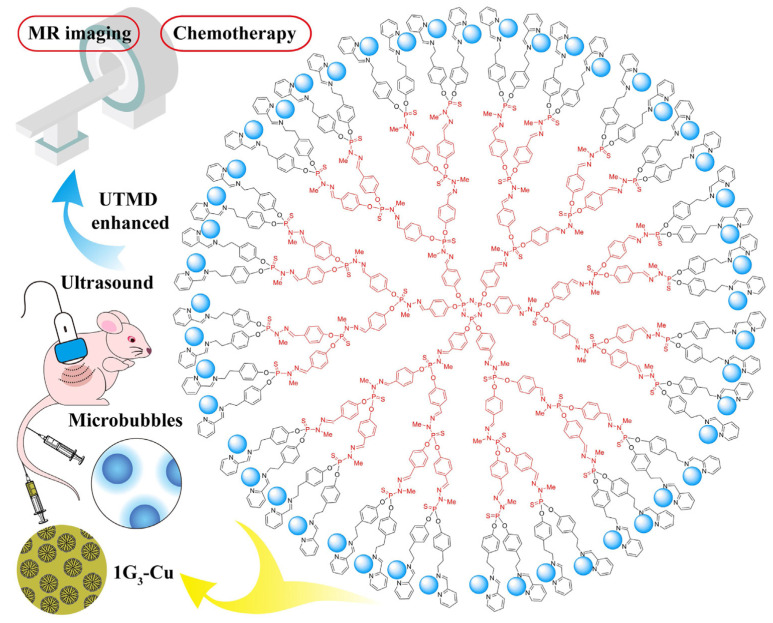
Schematic depiction of **1G3-Cu** to take down SW1990 tumors using the UTMD-enhanced MR imaging strategy. Reproduced with permission from reference [38], Elsevier.

To go further in the theragnostic realm to treat unmet medical need, Shi et al. tested **1G3-Cu** in vivo against aggressive tumors, including pancreatic SW1990 tumors, using non-invasive ultrasound-targeted microbubble destruction (UTMD) technology (Figure 3) [38]. The UTMD technique increases the tumor penetration of drugs or nanoparticles by inducing a sonoporation effect [41]. With the application of UTMD, **1G3-Cu** enabled enhanced magnetic resonance imaging of tumors. Without UTMD, **1G3-Cu** was distributed in the liver, lung and kidney, whereas with UTMD a higher accumulation of **1G3-Cu** was observed in SW1990 tumors. Importantly, **1G3-Cu** displayed good anti-cancer effects on day 14 by decreasing the tumor volume by around 62% (versus 35% without UTMD).

**Figure 4:**
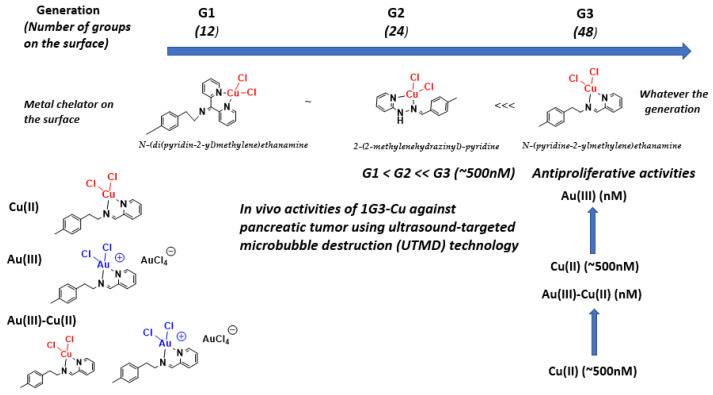
Global view of in vitro and in vivo antiproliferative activies of Cu(II)- and Au(III)-phosphorus dendrimers.

We also turned our attention to the influence of metal type on the anti-proliferative activities of the **1G3-Cu** metallodendrimer. **1G3-Au(III)**-based dendrimers complexed with the bidentate chelator *N*-(pyridin-2-ylmethylene)ethanamine have been tested against KB, HL60, EPC and MRC5 cell lines and the safety ratios evaluated. Notably, the replacement of Cu(II) by Au(III) boosted the anti-proliferative activity whichever tumor cell line was considered: IC_50_ = 470 nM for Cu(II) versus 7.5 nM for Au(III) against KB; and IC_50_ = 580 nM for Cu(II) versus 3.3 nM for Au(III) against HL60. The safety ratio related to normal MCR5 and EPC was 1–1.7 for Cu(II) compared to ~1.6–300 for Au(III). Several constructions of dual phosphorus dendrimers bearing Cu(II) and Au(III) on the same surface but in different ratios indicated that the presence of an Au(III) complex also induced anti-proliferative activities in the nM range over the µM range compared with Cu(II) alone. A global view of in vitro and in vivo anti-proliferative activities of Cu(II)- and Au(III)-phosphorus dendrimers is depicted in Figure 4.

**Figure 5:**
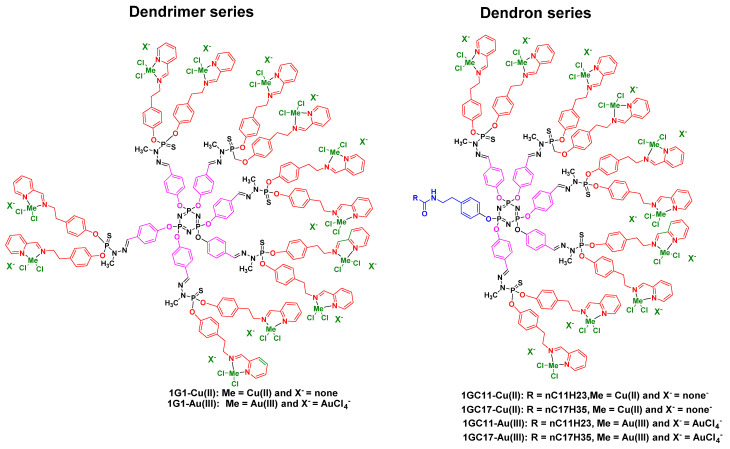
Schematic 2D chemical structures of dendrons **1GC_11_-Cu(II)**, **1GC_17_-Cu(II)**, **1GC_11_-Au(III)** and **1GC_17_-Au(III)**, dendrimers **1G1-Cu(II)** and **1G1-Au(III)**.

Taking inspiration from the potent anti-proliferative activity of the phosphorus dendrimers **1G3-Cu(II)** and **1G3-Au(III)** (*vide supra*), other studies were performed by Shi’s team regarding the development of first-in-class G1 Cu(II)- and Au(III)-metaled phosphorus dendrons using phenotypic screening [37]. Four dendrons bearing two different linear alkyl chains (C_11_H_23_ and C_17_H_35_) and ten *N*-(pyridin-2-ylmethylene)ethanamine groups to complex Cu(II) and Au(III) were fine-tuned and prepared in a five-step synthesis from hexachlorocyclotriphosphazene with good overall yields: **1GC_11_-Cu(II)**, **1GC_17_-Cu(II)**, **1GC_11_-Au(III)** and **1GC_17_-Au(III)** (Figure 5).

Dynamic light scattering studies showed that the hydrodynamic size of the dendron with the shortest alkyl chain (C11) was ~255 nm, which is smaller than that with the longest alkyl chain (C17) for the same metal (~298 nm).

The anti-proliferative activity of **1GC_11_-Cu(II)**, **1GC_17_-Cu(II)**, **1GC_11_-Au(III)** and **1GC_17_-Au(III)** was investigated against mouse breast adenocarcinoma 4T1 cells, which are highly tumorigenic and invasive, human breast adenocarcinoma MCF-7 cells, leukemia HL-60, human colon cancer HCT116 and, for safety reasons, against normal fibroblast NIH-3T3 cells and human fetal lung fibroblast MRC5 cells. Importantly, we compared the anti-proliferative activities of these four G1 metaled phosphorus dendrons versus the corresponding G1 dendrimers **1G1-Cu(II)** and **1G1-Au(III)** against leukemia HL-60 and human colon cancer HCT116 (Table 1). Dendron **1GC_11_-Cu(II**) displayed better anti-proliferative potency against 4T1, MCF-7 and HL-60 than dendron **C_17_-Cu(II).** The safety ratios of **1GC_11_-Cu(II)** (IC_50_ normal cell/IC_50_ tumor cell) are ~2.5, ~4, ~1 and ~1.7 against NIH-3T3/4T1, MRC5/4T1, NIH-3T3/MCF-7 and MRC5/MCF-7, respectively, whereas **1GC_17_-Cu(II)** showed safety ratios of ~4, ~3, ~1.5 and ~1, respectively against the same cell lines. As observed in the previous phosphorus dendrimer series (vide supra), the replacement of Cu(II) by Au(III) strongly improved the anti-proliferative activities by 1.5–5-fold in favor of Au(III) versus Cu(II) against 4T1, MCF-7 and HL-60. No clear structure–activity relationships (SARs) were observed for HCT-116. Thus, 1GC_11_-Au(III) and **1GC_17_-Au(III**) showed IC_50_ values of ~0.16–1.4 µM against 4T1, MCF-7 and HL-60 tumor cells. Dendrimers **1G1-Cu(II)** and **1G1-Au(III)** displayed about double the potency of the corresponding dendrons **1GC_11_-Cu(II)**, **1GC_17_-Cu(II)**, **1GC_11_-Au(III))** and **1GC_17_-Au(III)** against HL-60 and similar potencies against HCT116.

**Figure 6:**
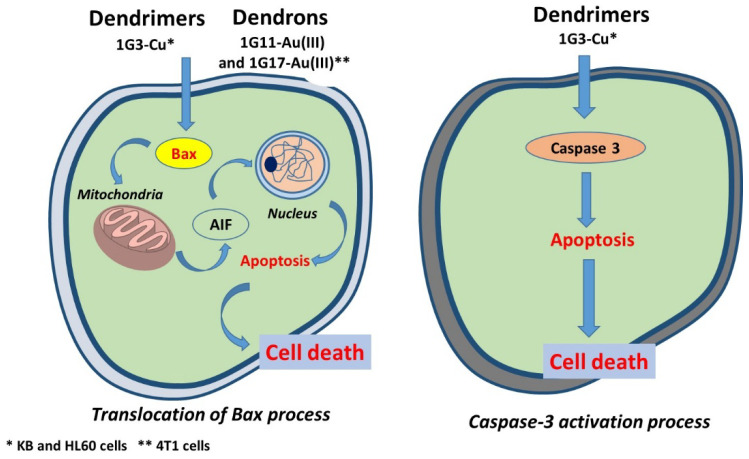
General view of the cell death pathway of phosphorus dendrons in comparison with the corresponding dendrimers.

Interestingly, the cell cycle and fluorescence microscopic images of the 4T1 cells clearly showed that the cell death pathway is related to the nature of the metal complexed by the dendrons. Thus, Cu(II)-metaled dendrons displayed a weak caspase-independent cell death pathway, whereas the corresponding dendrons with Au(III) showed a strong translocation of Bax into the mitochondria and the release of cytochrome c (CytoC). **1G3-Cu** dendrimer displayed strong translocation of Bax. Figure 6 depicts a general view of the cell death of phosphorus dendrons and dendrimers.

## 3. Polycationic Phosphorus Dendrimers

### 3.1. As Anti-Cancer Agents

**Figure 7:**
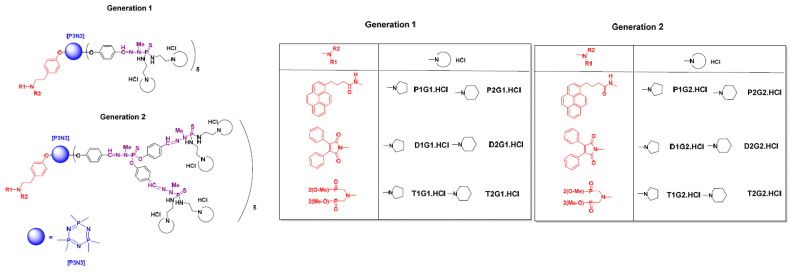
Chemical structures of polycationic phosphorus dendrons.

In the theranostic nanomedicine realm, the design and synthesis of first-in-class amphiphilic fluorescent phosphorus dendron-based micelles has been studied by the teams of Shi and Majoral [39]. As shown in Figure 7, 12 amphiphilic phosphorus dendrons bearing 10 protonated cyclic amino groups (G1) or 20 protonated cyclic amino groups (G2) and one hydrophobic chain carrying one fluorophore moiety were prepared and screened through phenotypic screening. Two cyclic amino groups were introduced on the surface (pyrolidino and piperidino moieties) and an aliphatic chain bearing three different groups, including fluorescent groups such as pyrene and maleimide and a non-fluorescent azabisdimethyl phosphonate group.

**Figure 8:**
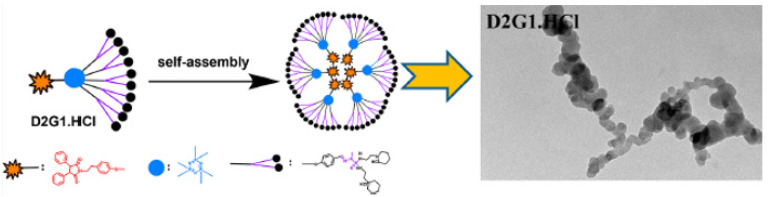
Self-assembly of **D2G1.HCl** amphiphilic dendrons to form nanostructured aggregates (micelles). Reproduced with permission from reference [39], American Chemical Society.

Previously, as markers of phosphorus dendrimers, several types of fluorophores have been used, such as pyrene, naphthol, anthracene, dansyl, diketone, phthalocyanine, maleimide, julolidine, rhodamine, fluorescein or fluorene derivatives [42,43]. Interestingly, these fluorescent amphiphilic phosphorus dendron-based micelles exhibited the capacity to aggregate in solution, mediated by hydrophilic/hydrophobic interactions, which promoted the formation of well-characterized polymeric micelles (Figure 8) [39]. 

The fluorescence spectra of amphiphilic pyrene dendrons showed excitation bands at 330 and 345 nm, which is consistent with characteristic luminescence peaks typically observed for pyrene monomers, whereas the UV–vis absorption spectra of amphiphilic maleimide dendrons showed two broad bands, one near 375 nm (aromatic groups of maleimide) and another around 284 nm, (phosphorus dendrons). Excitation bands around 380–390 nm and emission bands around 520–540 nm were present in the fluorescence spectra of amphiphilic maleimide dendrons. The critical micelle concentrations (CMCs) of the amphiphilic phosphorus dendrons in water are shown in Table 2.

Interestingly, for the pyrene series, unimolecular micelles were formed and remained unaltered regardless of dilution (above the CMC), and low differences between each micelle formed at different concentrations greater than the CMC were observed. The morphology and size of the aggregates of amphiphilic fluorescent phosphorus dendron-based micelles were evaluated using transmission electron microscopy. The sizes of the amphiphilic dendron-based micelles ranged from 200 to 500 nm, and increased with generation number. For the same generation, amphiphilic dendrons containing protonated pyrrolidinium end groups were slightly larger than those formed from amphiphilic dendrons with protonated piperidinium groups.

These phosphorus dendron-based micelles showed moderate to high (IC_50_ = 0.2–50 µM) anti-proliferative activities themselves against a panel of tumor cell lines, including lung carcinoma A549, breast carcinoma MCF7, breast carcinoma metastasis MDA-MB-231, prostate carcinoma PC3, brain glioblastoma U87-MG, acute promyelocytic leukemia HL60, chronic myelogenous leukemia K562, myelogenous leukemia K562R (doxorubicin resistant due to high P-glycoprotein expression) and colorectal carcinoma HCT116. One of the most interesting phosphorus dendron-based micelles is T2G2.HCl (G2), showing anti-proliferative activities against normal mouse fibroblast L929 cells, A549, MCF7, PC3, U87-MG, HL60, HCT116, K562, K562R and MDA-MB6231 tumor cell lines, with IC_50_ values of 0.2–2.6 µM (Table 3). Interestingly, **T2G2.HCl** (Table 3) displayed an IC_50_ of 2.47 µM against K562R, which is a tumor cell line that is doxorubicin-resistant due to high P-glycoprotein expression. Doxorubicin showed an IC_50_ of >50 µM against K562R.

These phosphorus dendron-based micelles can be used to efficiently perform traceable imaging, targeted delivery and selective therapy.

### 3.2. As Anti-Tuberculosis Agents

**Figure 9:**
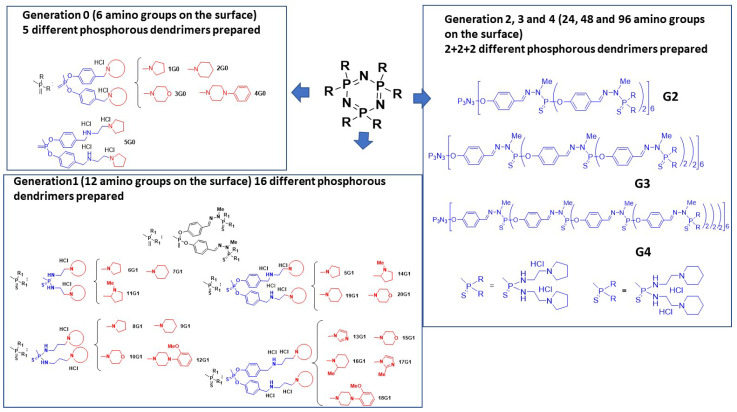
Chemical structures of prepared polycationic phosphorus dendrimers.

Tuberculosis is a contagious, airborne infectious disease caused most commonly by infection with various pathogen strains of mycobacteria, such as *Mycobacterium tuberculosis*. Tuberculosis represents a particularly significant burden to low- and middle-income countries, as current treatments are inadequate [44,45]. The specific structure and chemical composition of the mycobacterial cell wall hinders the entry of drugs, rendering many drugs and antibiotics ineffective, so that effective treatment remains difficult. Recently, an interesting review highlighting the development of nanoparticles in tuberculosis treatment was published [46]. Phenotypic screening of an original-designed polycationic phosphorus dendrimer library (G0–G4) against three bacterial strains was performed: attenuated *M. tuberculosis* H37Ra, virulent *M. tuberculosis* H37Rv and *M. bovis* BCG. The toxicity of these phosphorus dendrimers was evaluated against the kidney epithelial Vero cell lines. As shown in Figure 9, five polycationic phosphorus dendrimers (G0, 6 amino groups on the surface), sixteen polycationic phosphorus dendrimers (G1, 12 amino groups on the surface), two polycationic phosphorus dendrimers (G2, 24 amino groups on the surface), two polycationic phosphorus dendrimers (G3, 48 amino groups on the surface) and two polycationic phosphorus dendrimers (G4, 96 amino groups on the surface) were fine-tuned and prepared. These 27 phosphorus dendrimers have diverse types of amino groups on their surface, including pyrrolidinium, *N*-methyl-pyrrolidinium, piperidinium, *N*-methyl-piperidinium, morpholinium, imidazolinium, 2-methyl-imidazolinium, 1-phenyl-piperazinium and (2-methoxy)-1-phenyl-piperazinium. Several types of linkers have been introduced between the cyclotriphosphazene core ring and the diverse amino groups on the surface (Figure 9).

**Figure 10:**
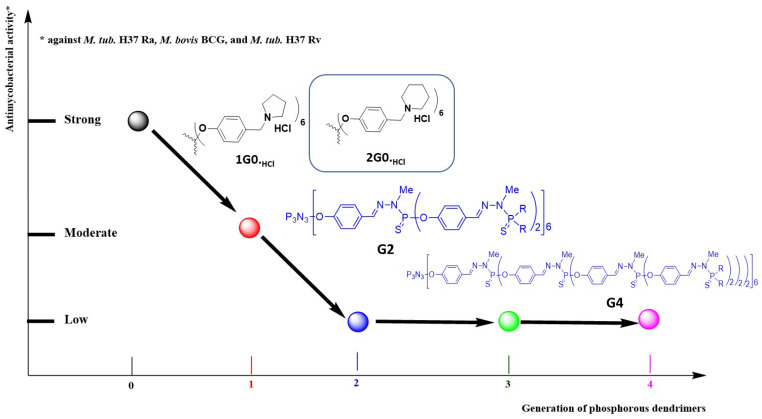
SARs between the generation of dendrimers (0–4) and their respective antimycobacterial activities.

Importantly, the most interesting is the G0 polycationic phosphorus dendrimers. Within this generation, antimycobacterial activities (minimum inhibitory concentration, MIC) against the three strains H37Ra, BCG and H37Rv are as follows: **1G0._HCl_** (MIC in µg/mL: 12.5 for Ra, 12.5 for BCG and 3.12 for Rv), **2G0._HCl_** (MIC in µg/mL: 25.0 for Ra, 25.0 for BCG and 3.12 for Rv), **3G0_,HCl_** (MIC = 6.25 µg/mL against Rv) and **5G0_,HCl_** (MIC in µg/mL: 50 for Ra and 100 for BCG). The two most potent compounds are **1G0._HCl_** and **2G0._HCl,_** and, interestingly, the safety index (CC_50_ against the Vero cell line divided by the respective MIC) is 16.02 versus Ra and 3.1 versus Ra for **2G0._HCl_** and **1G0._HCl_**, respectively. Figure 10 presents the SARs based on antimycobacterial activities among the prepared dendrimers between the generations of phosphorus dendrimers (G0–G4). Clearly, the increase of generation drastically decreased the anti-tuberculosis activities. Consequently, ‘poor’ SARs should be highlighted; however, the G0 polycationic phosphorus dendrimers were the most potent (six cyclic amino groups on the surface) and within this series, the introduction of a piperidine group on the surface produced the most potent and the safest phosphorus dendrimer.

As shown in Table 4, interestingly, **2G0._HCl_** showed significant activity against all the single drug-resistant *M. tuberculosis* strains, including the rifampicin-, isoniazid- and ethambutol-resistant strain H37Rv. In addition, in infected J774 A.1 cells, **2G0._HCl_** inhibited mycobacterial growth and intracellular survival, with an MIC of 6.12 µg/mL.

As **2G0._HCl_** demonstrated safe and potent in vitro antimycobacterial activity (vide supra), **2G0._HCl_** was evaluated for its in vivo efficacy in Balb/C mice. Two weeks of oral treatment with 33 or 50 mg/Kg **2G0._HCl_** (once-daily administration) significantly reduced the mean bacterial count in lung of infected mice by 1.0 log_10_ or ~1.5 log_10_ versus the untreated group. Interestingly, this in vivo result is even better than those obtained for the rifampicin-treated groups. Furthermore, **2G0._HCl_** showed better in vivo efficacy in comparison to ethambutol and rifampicin.

## 4. Conclusions and Perspectives

Medicinal chemists are like painters, they spread their ideas onto their palette in order to conceive new medicines according to their aesthetic and imaginative space and their background. This philosophy joins that of the famous painter Henri Matisse (1869–1954), who said ‘C’est l’imagination qui donne au tableau espace et profondeur (‘It is the imagination that gives the painting space and depth’) [47]. The versatility of phosphorus dendrimers and dendrons as drugs themselves has revealed fascinating properties in different therapeutic fields in nanomedicine [48]. Collectively, the major advantages of phosphorus dendrimers and dendrons, for instance, in comparison with the widely known PAMAM dendrimers, include: easily tunable surface synthesis, allowing the development of phosphorus dendrimers and dendrons as active drugs per se; higher lot-to-lot chemical stability, mainly for the low generations (G0, G1); good manufacturing practices for clinical use, mainly for the low generations (G0, G1); and several routes of administration. Currently, we are only on the emerged tip of the iceberg with respect to their huge possibilities as first-in-class drugs. It is at this level that medicinal chemists intervene with their imagination to create new drugs for unmet medical needs. In this concise review, we highlighted the salient in vitro and in vivo results for neutral metallo-dendrimers bearing Cu(II) or Au(III) salts on their surface as anti-cancer agents. Of note is that the increase of generation increases the anti-proliferative activities. An original mechanism of action (Bax activation) was clearly identified. Comparison of the anti-proliferative activities of metallo-dendrimers or metallo-dendrons bearing Cu(II) or Au(III) salts fully confirms the great interest in developing phosphorus dendrons as drugs with an original molecular mechanism of action (Bax activation). On the other hand, remarkably, first-in-class polycationic phosphorus dendrimers showed strong in vitro and in vivo anti-tuberculosis activity against *Mycobacterium tuberculosis* and represent a new chemical entity in the fight against tuberculosis. In a surprising manner, the G0 phosphorus dendrimers showed salient in vitro and in vivo anti-tuberculosis activities. We are convinced of the futuristic vision in nanomedicine of developing new dendrimers and dendrons in general, and phosphorus dendrimers and dendrons in particular, as first-in-class drugs of low generation. The advantages of low-generation phosphorus dendrimers and dendrons should be as follows: few chemical steps leading to G0 in high overall yield; high water solubility; strong chemical stability for more than 1 year; ability to be prepared under good manufacturing conditions in large quantities; ease of modifying the nature and the number of terminal groups to build diverse libraries; and cheap starting materials. An important part of our research was the use of phenotypic screening as a powerful tool for identifying new active chemical entities, in our case phosphorus dendrimers and dendrons, with an original mechanism of action reinforcing the concept of chemical space availability by the increase of affinity and selectivity towards their respective targets [49] using, for instance, dendrimers and dendrons (and biologics) versus small molecules or peptides [23]. In a nutshell, phosphorus dendrimers and dendrons, active per se, present a new strategy for the medicinal chemist in the development of new drugs, with much research still to be done and fundamental tenets to be discovered.

## Figures and Tables

**Table 1:** Anti-proliferative activities of **1GC11-Cu(II)**, **1GC17-Cu(II)**, **1GC11-Au(III)**, **1GC17-Au(III)**, **1G1-Cu(II)** and **1G1-Au(III)**.

Materials	Cell Lines						
		4T1	MCF-7	HL-60	HCT-116	NIH-3T3 *	MCR5 *
Dendrons	**1GC11-Cu(II)**	0.585+/−0.11	1.489+/−0.27	1.93+/−1.37	3.37+/−0.87	1.491+/−0.22	2.46+/−1.20
	**1GC17-Cu(II)**	1.025+/−0.09	2.755+/−0.286	2.65+/−0.65	3.03+/−0.254	4.075+/−0.65	2.66+/−0.64
	**1GC11-Au(III)**	0.164+/−0.04	0.286+/−0.07	1.33+/−0.25	2.09+/−0.23	0.468+/−0.12	2.71+/−0.71
	**1GC17-Au(III)**	0.339+/−0.06	0.802+/−0.15	1.34+/−0.30	4.44+/−0.87	0.132+/−0.06	3.18+/−0.27
Dendrimers	**1G1-Cu(II)**			1.00+/−0.20	3.26+/−0.71		
	**1G1-Au(III)**			0.65+/−0.04	2.90+/−0.87		

* Normal cell line. See reference [37] for detailed data.

**Table 2:** CMC (µM) of amphiphilic phosphorus dendrons in water.

Generation	Cyclic Amine	Pyrene Series 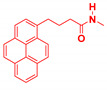	Maleimide Series 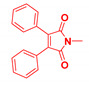	Azabisdimethyl Phosphonate Series 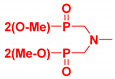
**1**	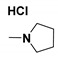	3.56	76.9	153.7
	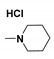	2.96	73.3	109
**2**	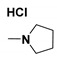	1.42	35.3	60
	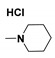	2.31	26.3	45.5

**Table 3:** Antiproliferative activities of **T2G2.HCl**.

Cell Lines	IC_50_s	Cell Lines	IC_50_s
L929 *	8.75+/−0.51	HL60	4.1+/−0.15
A549	0.36+/−0.06	HCT116	0.86+/−0.02
MCF7	2.89+/−0.17	K562	1.1+/−0.06
PC3	0.56+/−0.03	K562R	2.47+/−0.07
U87-MG	0.27+/−0.03	MDA-MB-231	0.31+/−0.01

* Normal cell line. See publication [39] for detailed data.

**Table 4:** Anti-TB activities of **2G0.HCl** against single drug-resistant Mtb strains.

Compound	*M. tuberculosis* H37Rv (µg/mL)	MTB Strain Resistant to			
		INH (µg/mL)	RIF (µg/mL)	ETB (µg/mL)	STR (µg/mL)
**2G0._HCl_**	3.12	6.25	6.25	6.25	6.25
RIF	0.04	0.04	>64	0.19	0.04
INH	0.04	>64	0.04	0.04	0.04
ETB	3.12	NT	NT	>64	0.78
STR	1.56	NT	NT	12.5	>64

RIF: Rifampicin, INH: Isoniaid, ETB: Ethambutol, STR: Streptomycin, NT: Not Tested.

## Sample Availability

Samples of the compounds are not available.
